# Identification and characterization of a novel SNAT2 (SLC38A2) inhibitor reveals synergy with glucose transport inhibition in cancer cells

**DOI:** 10.3389/fphar.2022.963066

**Published:** 2022-09-21

**Authors:** Gregory Gauthier-Coles, Angelika Bröer, Malcolm Donald McLeod, Amee J. George, Ross D. Hannan, Stefan Bröer

**Affiliations:** ^1^ Research School of Biological Sciences, Australian National University, Canberra, ACT, Australia; ^2^ Research School of Chemistry, Australian National University, Canberra, ACT, Australia; ^3^ The John Curtin School of Medical Research, Australian National University, Canberra, ACT, Australia

**Keywords:** amino acid transport system, amino acid metabolism, glutaminolysis, glucose transport, amino acid transport, BAY-876

## Abstract

SNAT2 (SLC38A2) is a sodium-dependent neutral amino acid transporter, which is important for the accumulation of amino acids as nutrients, the maintenance of cellular osmolarity, and the activation of mTORC1. It also provides net glutamine for glutaminolysis and consequently presents as a potential target to treat cancer. A high-throughput screening assay was developed to identify new inhibitors of SNAT2 making use of the inducible nature of SNAT2 and its electrogenic mechanism. Using an optimized FLIPR membrane potential (FMP) assay, a curated scaffold library of 33934 compounds was screened to identify 3-(*N*-methyl (4-methylphenyl)sulfonamido)-*N*-(2-trifluoromethylbenzyl)thiophene-2-carboxamide as a potent inhibitor of SNAT2. In two different assays an IC_50_ of 0.8–3 µM was determined. The compound discriminated against the close transporter homologue SNAT1. MDA-MB-231 breast cancer and HPAFII pancreatic cancer cell lines tolerated the SNAT2 inhibitor up to a concentration of 100 µM but in combination with tolerable doses of the glucose transport inhibitor Bay-876, proliferative growth of both cell lines was halted. This points to synergy between inhibition of glycolysis and glutaminolysis in cancer cells.

## Introduction

Amino acid homeostasis in cancer cells requires the import of essential and conditionally essential amino acids to sustain protein and nucleotide biosynthesis and to keep the mechanistic Target of Rapamycin Complex 1 (mTORC1) in an activated state (Broer, 2020). The import of amino acids into cells is mediated by an array of amino acid transporters, which can be functionally classified as loaders, harmonizers and controllers (Gauthier-Coles et al., 2021). Loaders mediate the net accumulation of amino acids by Na^+^-amino acid cotransport, such as SNAT1 (SLC38A1), SNAT2 (SLC38A2), and SNAT4 (SLC38A4). But they can also be electrogenic uniporters such as the cationic amino acid transporter CAT1 (SLC7A1). The loaded amino acids are then exchanged for other, often essential, amino acids via antiporters such as LAT1 (SLC7A5), ASCT2 (SLC1A5), and y^+^LAT2 (SLC7A6), which harmonize amino acid pools inside the cell. This harmonization allows cells to maintain a balanced mix of amino acids for homeostasis (Gauthier-Coles et al., 2021). The system has inbuilt redundancy to avoid complete reliance on any single transporter (Broer, 2020). This was illustrated by a systematic CRISPR-Cas9 drop-out screen (Behan et al., 2019) by the Sanger institute showing that dropout of only a few amino acid transporters caused a significant loss of fitness (defined as genes required for cell growth or viability) in >800 cancer cell lines. This included the widely expressed amino acid exchanger LAT1 (reduced fitness in 400 out of 811 cancer cell lines), SNAT2 (reduced fitness in 24% of cancer cell lines and 75% of cancer types) and CAT1 (reduced fitness in 18% of cell lines and 80% of cancer types). These results emphasize the role of loaders such as SNAT2 and CAT1 in cancer cells for the supply of essential amino acids.

SNAT2, in conjunction with SNAT1, has a unique role in providing net glutamine for the glutaminolysis pathway (Broer et al., 2016; Broer et al., 2019). In addition, they maintain a cytosolic amino acid pool at elevated concentrations. SNAT2, due to its transceptor function, is also an activator of mTORC1 (Pinilla et al., 2011; Fairweather et al., 2021). These effects are likely to contribute to the reduced fitness in SNAT2ko cells noted above. Accordingly, the transporter has received increasing attention as a factor in cancer cell growth (Jeon et al., 2015; Morotti et al., 2019; Huang et al., 2020; Parker et al., 2020; Hua et al., 2021; Kandasamy et al., 2021; Morotti et al., 2021). In addition, it has been proposed that SNAT2 promotes stem-cell-like characteristics in gastric cancer cells (Nie and Cai, 2021).

SNAT2 is a highly inducible transporter, the activity of which is barely detectable when cells are grown in amino acid-rich media (Broer et al., 2019; Menchini and Chaudhry, 2019). However, its surface expression can be upregulated many-fold by incubating cells in an amino acid-free medium for several hours (Broer et al., 2019), which increases relocation from intracellular stores and *de novo* synthesis (Hyde et al., 2001; Gaccioli et al., 2006).

As cellular entry and exit points, transporters are closely linked to metabolism. Two of the main metabolic rearrangements in cancer cells are the Warburg effect (Vander Heiden et al., 2009) and glutamine addiction (Wise and Thompson, 2010). The Warburg effect refers to a glycolytic pathway largely converting pyruvate into lactate instead of its transfer into mitochondria. This is complemented by the partial conversion of the TCA cycle into a linear pathway that does not require pyruvate, i.e. glutaminolysis (Broer, 2020). This allows shunting of glutamine into the production of nucleobases via the formation of aspartate and glutamate (Newsholme et al., 1985). Glutamate, in turn, can be used as an exchange substrate to acquire cystine for the maintenance of glutathione levels (Nilsson et al., 2020). The link between glutaminolysis and lactate-producing glycolysis points to a possible synergy when inhibiting both pathways. Both pathways have been targeted for instance through blocking of glucose transport via GLUT1 using inhibitor Bay-876 (Siebeneicher et al., 2016) or blocking deamination of glutamine by the glutaminase inhibitor CB-839 (Gross et al., 2014).

Current approaches to inhibit SNAT2 *in vitro* rely on the amino acid analogue N-methyl-aminoisobutyric acid (MeAIB), which was developed after recognizing that alanine transport in Ehrlich ascites tumor cells was receptive to amino-methylated substrates in contrast to most other amino acid transporters (Christensen et al., 1965). However, this compound cannot discriminate between SNAT1 and SNAT2. While betaine discriminates between SNAT1 and SNAT2 it has a very low affinity and inhibits other transporters (Nishimura et al., 2014). Development of new amino acid transport inhibitors (Broer, 2018) has relied on a mix of approaches including the synthesis of substrate analogues (Esslinger et al., 2005; Albers et al., 2012; Schulte et al., 2018), experimental and *in silico* small scale screening (Geier et al., 2013; Pochini et al., 2014; Colas et al., 2015; Danthi et al., 2018; Yadav et al., 2020) and high-throughput screening (HTS) (Benjamin et al., 2005; Kutchukian et al., 2017). While medicinal chemistry of substrate analogues is straightforward, it bears the risk that lead compounds might be non-specific and prone to competition by substrates. Compound library screening approaches in the presence of saturating substrate concentrations are more likely to circumvent both problems. Particularly useful probes for transporter HTS are membrane potential sensitive dyes (Benjamin et al., 2005; Cheng et al., 2017). The commercially available FLIPR (Fluorometric imaging plate reader) membrane potential assay (FMP) makes use of anionic dyes which can penetrate into the membrane. The negative charge repels the dyes from negatively polarized membranes, allowing dye to penetrate the membrane upon depolarization (Bashford et al., 1979). The fluorescence of the dye increases in the membrane environment, while extracellular dye is quenched by a second dye that absorbs the fluorescence. When fluorescence is recorded from the bottom of a well only the dye penetrating into the membrane generates a signal, which increases during depolarization.

In this study we have used a FLIPR membrane potential assay (FMP) to identify a novel class of SNAT2 inhibitors. These inhibitors were tested on several cancer cell lines and found to synergize with the glucose transporter (GLUT1) inhibitor Bay-876.

## Materials and methods

### Cell culture

The cell lines used in this study (ovarian adenocarcinoma SKOV-3, pancreatic adenocarcinoma HPAFII, breast adenocarcinoma MDA-MB231, acantholytic squamous cell carcinoma HCC 1806) were obtained from the American Type Culture Collection (ATCC).

All cell lines were cultured in Dulbecco’s Modified Eagle Medium/Nutrient Mixture F-12 (DMEM/F-12) supplemented with 10% heat-inactivated Foetal Bovine Serum (FBS), 2 mM glutamine and antibiotics/antimycotics (always 10 U/mL penicillin and 10 μg/ml streptomycin) (Broer et al., 2019). Cells were cultured up to 30 passages before being replaced with an earlier-passage aliquot of cells. All cell lines were maintained at 37°C in a humidified atmosphere containing 95% air and 5% CO_2_ and were trypsinised with 0.25% trypsin-EDTA when confluency reached >90%.

### Genome editing of SNAT2

To delete the SNAT2 (SLC38A2) gene in modified HCC1806 cells (clone C10 nullizygous for ASCT2 (Broer et al., 2019)), the GeneArt CRISPR Nuclease Vector with OFP Reporter Kit (Invitrogen; A21174) was employed and complementary 19–20 bp long single-stranded oligonucleotides with overhangs were designed to produce a guide RNA (gRNA) for the Cas9 nuclease that would target exon 7 of the SNAT2 gene. Colonies were analysed by sequencing using the U6 primer included in the GeneArt CRISPR Nuclease Vector with OFP Reporter kit. Plasmids that contained the correct CRISPR nuclease construct were then used to transfect HCC1806 cells with Lipofectamine 2000 (Invitrogen). Prior to transfection, medium was replaced with 2 ml of fresh DMEM/F-12 (supplemented with 10% heat-inactivated FBS and 2 mM glutamine). Plasmid DNA (4 µg) and 10 µL of Lipofectamine 2000 were incubated separately for 5 min at room temperature in 500 µl of Opti-MEM (Invitrogen) before being combined and incubated for a further 30 min at room temperature. The DNA-Lipofectamine complex was then added to the cells in a dropwise manner and allowed to be absorbed over 6 h at 37°C, after which the medium was renewed with fresh DMEM/F-12. Cells were checked for red fluorescence using an EVOS FL cell imaging system (Thermo Fisher Scientific) 48–72 h post transfection. Subsequently, cells were detached using 0.25% Trypsin EDTA. After harvesting and washing thrice with PBS (pH 7.4), cells were resuspended in PBS (pH 7.4) supplemented with 5 mM glucose and 1% dialysed and heat inactivated FBS and passed through a 100 μm cell strainer. The cell filtrate was then pelleted once more and resuspended in the same buffer. Cells were sorted using an ARIA II FACS machine and individually dispensed into the wells of a 96-well plate. Nullizygous clones were identified using a series of assays including the FLIPR membrane potential assay, western blotting and sequencing.

### Surface biotinylation and western blotting

Plasma membrane proteins were isolated from mammalian cells for western blot analysis using surface biotinylation and streptavidin coated beads. Cells were grown in 35 mm or 60 mm dishes until confluent, washed three times with modified PBS (supplemented with 1 mM CaCl_2_ and 0.5 mM MgCl_2_; pH 8.0) and incubated with modified PBS containing 0.5 mg/ml EZ-Link Sulfo-NHS-LC-Biotin (Thermo Scientific; 21335; pH 8.0) at RT for 30 min. Excess biotinylating agent was quenched and removed by washing the cells thrice with modified PBS containing 100 mM glycine (pH 8.0) and homogenised with 1 ml of lysis buffer (150 mM NaCl; 20 mM Tris; 1% Triton X-100; pH 7.5) containing cOmplete Protease Inhibitor Cocktail (Roche). The lysate was collected in a microcentrifuge tube and incubated on ice for 2 h with occasional inversion, before centrifuging the tube at top speed for 5 min. Of the supernatant 10 µl was used for protein determination. Volumes, equivalent by protein mass, of each lysate were added to 30 µl of High-Capacity Streptavidin Agarose (Thermo Scientific) resin and incubated overnight at 4°C with rotation. The following day, each tube was centrifuged at top speed for 5 min and the supernatant removed. The agarose resin was washed four times with fresh lysis buffer and prepared for SDS-PAGE.

Protein matrices from whole cell lysate and surface biotinylated samples were separated using SDS-PAGE. To prepare the samples, 12.5 µl of 4X Bolt LDS Sample Buffer and 5 µl of 10X Bolt Sample Reducing Agent (Life Technologies) were added to 30 µl of lysate or streptavidin agarose slurry diluted in water to the appropriate final concentration of protein. SeeBlue Plus2 or Novex Sharp pre-stained protein standards (Invitrogen; LC5925 and LC5800) were used for size comparison. Following gel electrophoresis, proteins were transferred onto a 0.45-micron Amersham Protran nitrocellulose membrane (GE Healthcare) using a Mini-Trans Blot unit (Bio-Rad). The blot was then incubated in 7.5% (w/v) skim milk powder in PBS-Tween (0.1% Tween-20; pH 7.4) over night at 4°C. Between blocking and antibody incubations, blots were washed thrice with PBS-Tween for 5–10 min at a time.

A list of antibodies, their dilutions and source, is provided in Table 1. Primary antibodies were incubated with blots overnight, while secondary antibodies were incubated for 2–6 h. To visualise protein, either the SuperSignal West Pico PLUS or the SuperSignal West Femto Maximum Sensitivity chemiluminescent substrates (Thermo Scientific; 34577 and 34094) were added dropwise to the blots.

**TABLE 1:** Antibodies and their source and dilutions.

Primary antibodies	Source	Identifier	Dilution
AATs			
SNAT1 (SLC38A1)	UC Davis/NIH NeuroMab Facility	N104/37	1:2000
SNAT2 (SLC38A2)	Abcam	ab90677	1:1000
Control			
Sodium Potassium ATPase	Abcam	ab76020	1:5000
Secondary antibodies			
Goat anti-rabbit IgG, HRP-linked	Cell Signaling	7074	1:2000–1:10,000
Horse anti-mouse IgG, HRP-linked	Cell Signaling	7076	1:2000–1:4000

All blots were probed first for the protein(s) of interest and lastly for a housekeeping protein (Na^+^/K^+^-ATPase). To remove membrane-bound antibodies from the previous probe, blots were incubated at 70°C with erasing buffer (2% w/v SDS; 62.5 mM Tris; and 0.7% v/v β-mercaptoethanol; pH 6.8) for 20 min with agitation. The blots were then washed with PBS-Tween and blocked for at least 2 hours with 7.5% skim milk in PBS-Tween before reprobing.

### Amino acid transport assay

Functional characterisation of plasma membrane transporters in human cancer cells was determined at 37°C using an array of radiolabelled amino acid substrates. Modified Hanks HEPES-buffered salts solution (mHBSS; 137 mM NaCl; 5.4 mM KCl; 2.7 mM K_2_HPO_4_; 1 mM CaCl_2_; 0.5 mM MgCl_2_; 0.44 mM KH_2_PO_4_; 0.4 mM MgSO_4_; 5 mM D-glucose; 5 mM HEPES; pH 7.4) was used to wash and incubate cells where sodium dependent transport was measured, while *N*-methyl-*D*-glucamine (NMDG) and potassium salts substituted sodium in NMDG mHBSS to measure sodium independent uptake. For the assay, cells were seeded in 35 mm dishes and, once confluent, were washed thrice with warm mHBSS solution and incubated with mHBSS containing an 100 µM amino acid at a final specific activity of ≥2000 cpm/nmol (U-[^14^C]amino acids were from PerkinElmer). After 6 min, cells were washed thrice with ice-cold mHBSS, lysed with 500 µl of 0.1 M HCl, detached with a cell scraper and triturated. A 400 µl aliquot was used for scintillation counting, while the remainder was saved for protein mass quantification by Bradford assay. Transport rates were normalised to the protein content of the culture dish.

### Growth assay

Cells were seeded on 96-well flat bottom plates at approximately 3000 cells/well and incubated in growth medium (DMEM/F12 supplemented with 10% FBS, 2 mM glutamine) for 24 h. The next day, the growth medium was aspirated and exchanged with 300 μL/well of growth medium containing compounds at the indicated concentration. For live cell analysis, plates were placed in the IncuCyte system (Essen Bioscience), which was housed in a cell incubator (37°C, humidified atmosphere composed of 95% air and 5% CO_2_) and confluence measured at 6-h intervals or at the assay endpoint when untreated control cells reached a confluency >90%. Antibiotics (10 U/mL penicillin and 10 μg/ml streptomycin) were included in the treatment solutions.

### FLIPR membrane potential (FMP) assay

The FLIPR Membrane Potential Blue Explorer Kit (Molecular Devices) was used to characterise inhibitors and to optimise a high-throughput compound screen. Cells were seeded at approximately 60,000 cells/well on a 96-well plate and allowed to attach and grow overnight in a cell incubator. The next day, the cells were washed once with mHBSS and incubated in a starvation medium (composed of mHBSS with 1% dialysed heat-inactivated FBS, 23 mM NaHCO_3_, 10 U/ml penicillin and 10 μg/ml streptomycin) for 24 h. Cells were then washed once with mHBSS and incubated with 50 µl of the mHBSS-resuspended FMP assay dye and 50 µl of mHBSS (with or without inhibitors) and incubated at room temperature for 30 min. The plate was then placed in an EnSpire Multimode plate reader (PerkinElmer) and four sequential measurements of fluorescence were recorded as a baseline reference. Immediately after the baseline measurements were taken, 25 µl of 5 mM glutamine dissolved in mHBSS was injected into each well and the plate was shaken orbitally for 5 s before a series of consecutive fluorescence measurements were taken. The excitation wavelength was set to 530 nm and the emission was detected at 565 nm. The Δ fluorescent signal reported in the figures was taken 30 s after substrate addition. After the assay was complete, the liquid content of the plate was carefully removed, and the cell monolayer inspected to ensure it remained intact throughout the course of the assay.

### High-throughput compound screen (HTS)

For the high-throughput compound screen, the FMP assay was adapted into a 384-well format within the ANU Centre for Therapeutic Discovery. HCC1806 cells were selected due to their superior adhesive properties after 24 h amino acid depletion. Compounds Australia stores compounds under robust environmental conditions and supplies them in assay ready plate format. We screened 33,934 compounds from the Collection Scaffold Library. Cells (10,000/well) were seeded into Corning 384-well black walled plates (CLS3985; Sigma-Aldrich) using a Multidrop Combi liquid dispensing robot (Thermo Fisher) in a 40 µl volume. Once seeded, cells were allowed to grow in a microplate incubator (Liconic) for 24 h at 37°C (humidified atmosphere with 95% air and 5% CO_2_). Subsequently, plates were washed thrice with 50 µL of mHBSS using a 405 TS washer (BioTek). Then, 50 µl of starvation medium was added and cells were incubated again for 24 h.

Assay-ready compound plates (384 well plates containing compound or DMSO controls) were then prepared prior to addition to cells. In addition to the supplied compounds, 5 µl γ-glutamyl-hydroxamate (GHX, 60 mM dissolved in mHBSS, serving as an inhibitor control) was added into inhibitor control wells using a JANUS G3 robot (PerkinElmer). Subsequently, 25 uL of mHBSS was dispensed into all wells of the 384 well plate using a Multidrop Combi. All samples were then mixed using the JANUS G3 robot and 15 µl dispensed on top of the cell monolayer to reach a final concentration of 10.05 µM.

The FMP dye was solved freshly with mHBSS, 20 µl of which was deposited into each well using the Multidrop Combi, and the cells were incubated at room temperatur for 30 min. Each plate was then placed in an EnVision microplate reader (PerkinElmer) and a single baseline measurement was taken for each well (bottom reads excitation at 531, emission at 579 nm). The plate was then transferred to the Multidrop Combi and 10 µl/well of 5 mM glutamine was dispensed into each well. After linearly shaking for 5 s, microplates were returned to the EnVision microplate reader for a single post-injection measurement per well. The change of fluorescence was measured after 60 s.

### Docking

Molecular docking was carried out using UCSF Chimera (version 1.1.6) (Pettersen et al., 2004) with AutoDock Vina as the docking module. The receptor was a SNAT2 homology model based on the *Danio rerio* Slc38A9 structure 7 kgv.2. A as provided by the Swiss Model database. The ligand was downloaded from PubChem and converted to pdb format prior to docking.

### Data analysis

IC_50_ were calculated using equation

$$y=y_{\max}\left(1-\frac{x}{x+{IC}_{50}}\right)$$

Growth curves were generated using the Gompertz function of OriginPro software:

$$y={ae}^{{-e}^{\left(-k\left(x-x_{c}\right)\right)}}$$

Where *a* is the maximum confluence or upper asymptote, *k* is the growth rate coefficient, *xc* is the time point at which 50% confluence is reached (inflection point) and *e* is Euler’s number. Cell growth data are shown as % confluence.

Synergism between drug combinations was quantified using ZIP synergy scoring as calculated by SynergyFinder (Yadav et al., 2015).

Z′ scores were calculated from the controls onboard each of the 384-well plates assayed in the primary and secondary screen as described by Zhang et al., (Zhang et al., 1999). For the control inhibitor γ-glutamyl-hydroxamate (GHX) a Z’ score of 0.717 was determined, which was a sufficient signal for a HTS.

The number of independent biological replicates used to generate data are indicated in the figure legends.

### Assay of glycolytic activity

Glycolytic activity was measured by detecting extracellular lactate levels over a 48-h time course. HCC1806 and HPAFII cells were seeded on a 24-well plate at approximately 500,000 and 700,000 cells/well, respectively, and incubated in growth medium overnight. The following day, the growth medium was aspirated and each well was washed once with growth medium and 1.3 ml of the treatment solution was added to begin the time-course. In addition to sampling the medium at the start of the experiment, 50 μL was collected from each well at the following time points: 9, 24 and 48 h. Samples were stored at −20°C until further use. Lactate was detected by an enzymatic assay, which consisted of combining in microcentrifuge tubes: 5 μl of sample, 45 μl of water; 25 μl of 40 mM NAD^+^; 421 μl of buffer (1M glycine, 600 mM hydrazine sulfate, 5.6 mM EDTA; adjusted to pH 9.5 with sodium hydroxide); and 4 μL of L-lactate dehydrogenase solved at 300U/mL (3.33 mg/ml). The tubes were briefly vortexed and incubated for 15 min before transferring 200 μL of the mixture to a 96-well plate. The calibration curve spanned final concentrations from 5 to 500 μM. Absorbance was measured at 340 nm to detect NADH. Lactate production was normalised to total cellular protein per well as measured by the Bradford assay.

### Amino acid analysis

Amino acids were analyzed with an Orbitrap Q-Exactive Plus coupled to an UltiMate 3000 RSLCnano system (Thermo Fisher) as described previously (Gauthier-Coles et al., 2021).

## Results

### Optimization of SNAT2 expression and assay conditions

**FIGURE 1:**
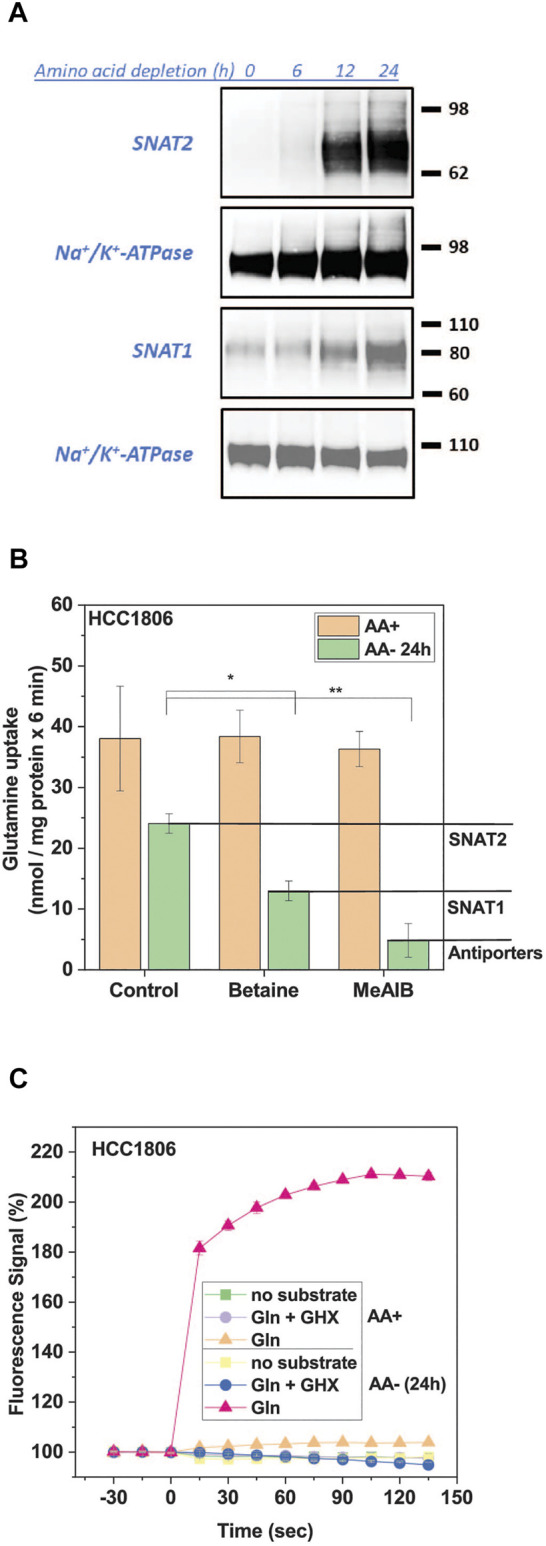
Induction of SNAT1 and 2 activity by amino acid depletion. HCC1806 cells were grown in complete growth medium or incubated in glucose-supplemented HEPES buffered Hanks’s buffered salt solution (mHBSS) for 24 h to induce amino acid transporter expression. **(A)** Surface expression of SNAT1 and SNAT2 in HCC1806 cells starved of amino acids over 24 h. Protein was separated by SDS-PAGE and analysed by western blotting. Molecular weight references are expressed in kDa, Na/K-ATPase was detected as a loading control. The blots shown are representative (n = 3). **(B)** Glutamine uptake in HCC1806 cells pre-incubated in growth medium (AA+) or starvation medium (AA-) for 24 h. Net transport rates were measured for 6 min at a substrate concentration of 100 µM in mHBSS. Betaine specifically inhibits SNAT2 and MeAIB is a SNAT1/2 inhibitor. Allocation of uptake activity to different transporters is indicated by the lines and labelling in the right margin. The transport rate of the background control, where radiolabelled glutamine was incubated in the presence of 10 mM of its unlabelled form, was subtracted from each transport rate. Data are shown as mean ± STD (n = 3). **(C)** Glutamine uptake was detected by using the FMP assay in HCC1806 cells pre-incubated in growth medium (AA+) or starvation medium (AA-) for 24 h. A time-course of the FMP assay is shown. HCC1806 cells were incubated with the FMP dye for 30 min before baseline measurements were taken every 15 s. Glutamine (1 mM final concentration) was introduced at t = 0. Gamma-glutamylhydroxamate (GHX) was used as an inhibitor control. Data are shown as the mean ± STD (n = 3). Significant differences from the control are indicated by * (*p* < 0.05), **(*p* < 0.01).

SNAT2 mediates the electrogenic Na^+^-dependent uptake of small and polar neutral amino acids in all known cell lines (Broer and Broer, 2017). The depolarization of cells upon exposure to substrate can be used as a signal for its transport activity. Before initiating the high-throughput screen we optimized the signal for the FMP assay. We selected HCC1806 triple negative breast cancer cells (Broer et al., 2019), which show robust adherence even after amino acid starvation. In HCC1806 cells SNAT2 expression was barely detectable after 6 h of incubation in amino acid-free media but was readily detected by surface biotinylation between 12 and 24 h (Figure 1A). Surface expression of SNAT1, which was already detectable in full growth medium also increased upon amino acid starvation (Figure 1A). This was confirmed by analysis of [^14^C]glutamine uptake in fed and amino acid-depleted cells (Figure 1B). Previous analysis of [^14^C]glutamine uptake in HCC1806 cells showed that it was mainly mediated by SNAT1, SNAT2, and the antiporters ASCT2, LAT1 and LAT2 (Broer et al., 2019). The incubation in amino acid-free medium not only increased SNAT2 activity, but also reduced ASCT2, LAT1 and LAT2 activity because antiporters rely on intracellular substrates to be active, which were depleted. Thus, although total glutamine uptake was reduced after amino acid depletion, induction of SNAT1 and SNAT2 was readily detectable by an increase of the fraction of transport that was sensitive to MeAIB (Figure 1B). In fed cells, MeAIB barely inhibited the uptake of glutamine, while in depleted cells glutamine uptake was reduced by 80% (Figure 1B). Betaine inhibited glutamine uptake by 46% suggesting that SNAT1 and SNAT2 were of similar activity in amino acid depleted cells.

The induction of SNAT1/2 could be readily detected by using the FMP assay (Figure 1C). Only SNAT1 and SNAT2 are electrogenic transporters that can contribute to the FMP assay signal. ASCT2, LAT1, and LAT2 are antiporters that do not depolarize cells upon substrate addition (Fairweather et al., 2021). This provides a background-free signal for inhibitor screening and characterization. In fed cells the contribution of SNAT1/2 to glutamine uptake was minimal and consequently no signal was detected when using the FMP assay to detect membrane depolarization (Figure 1C). In amino acid depleted cells, by contrast, glutamine generated a strong signal, which plateaued over time (Figure 1C).

**FIGURE 2:**
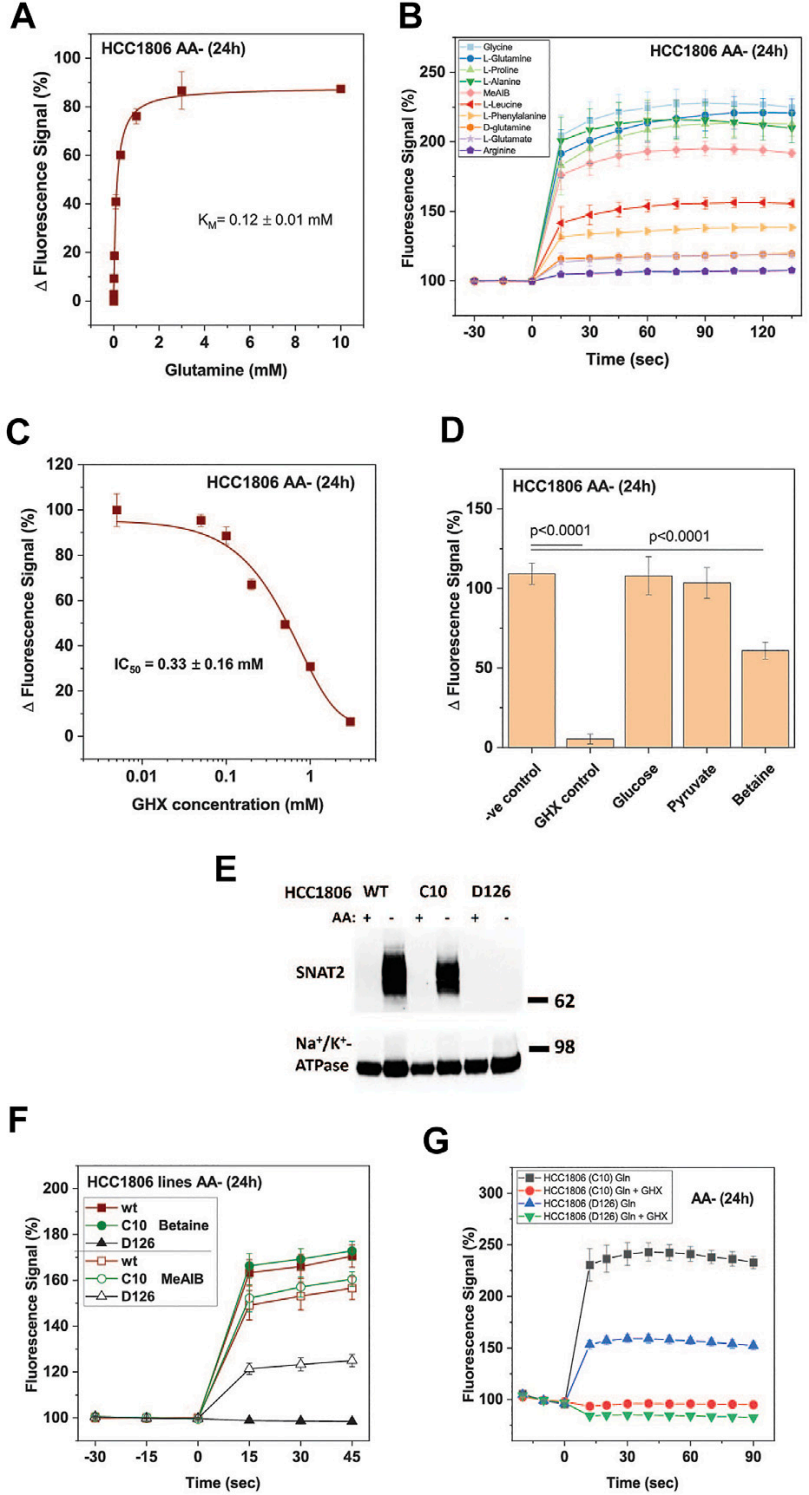
Optimization of HTS conditions to identify SNAT1 and 2 inhibitors. HCC1806 cells were incubated in glucose-supplemented Hanks’s buffered salt solution (mHBSS) for 24 h to induce amino acid transporter expression. **(A)** The FMP assay was used to determine kinetic parameters of glutamine transport. The plateau of the FMP signal was plotted against the glutamine concentration (n = 3). **(B)** Time course of the FMP signal for different amino acids at a final concentration of 1 mM (n = 3). **(C)** IC_50_ curve of GHX inhibition of glutamine induced membrane depolarisation using FMP assay. Data are plotted as mean ± STD (n = 3) percentage change between post-injection and baseline fluorescence measurements. **(D)** Starved HCC1806 cells were assayed on a 384-well plate using two non-inhibitors of SNAT2, glucose and pyruvate, and two low-affinity substrates, betaine and pyruvate (all at 10 mM). Depolarisation was elicited with 1 mM glutamine. Negative (−ve) control corresponds to pre-incubation of cells in FMP dye and plain mHBSS. The values shown correspond to the signal change after the injection of glutamine, expressed as a percentage of the baseline reading (n = 19). **(E)** Western blot of surface biotinylated protein detecting SNAT2 in HCC1806 C10 cells (ASCT2ko) and D126 (ASCT2ko, SNAT2 ko) cells starved of amino acids for 0 h (AA+) and 24 h (AA-). The Na^+^/K^+^-ATPase is shown as a loading control. **(F)** The FMP assay was used to compare SNAT1 and SNAT2 activity in wildtype HCC1806 cells, clone C10 (ASCT2ko) clone D126 (ASCT2ko, SNAT2 ko) after a 24-h amino acid starvation. Betaine was used as a SNAT2-specific substrate, MeAIB as a substrate for both transporters. **(G)** The FMP assay was used to measure SNAT2 activity in clone D126 (ASCT2ko, SNAT2 ko) compared to the parental C10 cell line (ASCT2ko) after 24-h amino acid starvation. Glutamine, at a final concentration of 1 mM was used to depolarise the membrane of cells. GHX was included as an inhibitor of SNAT1/2. Data are shown as the mean fluorescence signal normalised to the mean pre-injection signal ± STD (n = 6). Abbreviations: GHX (L-glutamic acid γ-monohydroxamate).

The signal was concentration dependent resulting in a K_M_ = 0.12 ± 0.01 mM for glutamine (Figure 2A), which is similar to K_M_-values measured for the isolated SNAT1 and SNAT2 transporters (Yao et al., 2000; Albers et al., 2001; Mackenzie et al., 2003). The substrate specificity was consistent with that of SNAT1/2 with alanine, glycine, glutamine, and proline providing the strongest response (Figure 2B). MeAIB gave a weaker signal consistent with its lower k_cat_. Leucine and phenylalanine, which are poor substrates of SNAT2 but not SNAT1 (Mackenzie et al., 2003; Fairweather et al., 2021), also gave a weak signal in the FMP assay. For the HTS 1 mM glutamine was used as a substrate to give a robust signal. As an inhibitor control for the HTS, we used γ-glutamyl-hydroxamate (GHX), a glutamine analogue, which blocks a variety of glutamine transporters such as ASCT2, SNAT1, SNAT2, SNAT4 and SNAT5 (Broer et al., 2016) and completely blocked the signal with an IC_50_ of 0.33 ± 0.16 mM (Figure 2C). GHX was used at a concentration of 6 mM throughout the screen. At this concentration the signal was reduced by 92% (*p* < 0.0001) (Figure 2D). The application of betaine (10 mM), a specific inhibitor of SNAT2, confirmed that both SNAT1 and SNAT2 contributed to the overall signal. We accepted this ambiguity as it would allow for the detection of inhibitors of SNAT1, SNAT2 or both transporters. Amino acid unrelated metabolites, such as glucose (10 mM) and pyruvate (10 mM) did not influence the signal (Figure 2D).

The contributions of SNAT1 and SNAT2 were confirmed using a SNAT2 knock-out HCC1806 clone (D126) and comparing it to the parental ASCT2 knock-out cell line (C10) that we have previously used to analyze the role of ASCT2 in cancer cells (Broer et al., 2019). The absence of SNAT2 in clone D126 was confirmed by western blotting (Figure 2E), while the parental line C10 and wildtype HCC1806 cells showed strong expression of SNAT2 upon amino acid depletion. Consistent with the western blot data, the SNAT2-specific substrate betaine (10 mM) generated a strong FMP signal in HCC1806 wildtype and in HCC1806-C10 cells (ASCT2ko) but not in HCC1806-D126 cells (ASCT2ko, SNAT2ko) (Figure 2F). MeAIB (10 mM), a substrate of SNAT1 and SNAT2, generated a similar signal in wild-type and C10 cells, but a much smaller signal (42%) in D126 cells consistent with expression of SNAT1 only (Figure 2F). Addition of glutamine (1 mM) to C10 cells resulted in a strong signal, which was fully blocked by GHX (Figure 2G). In D126 cells the glutamine signal was reduced to 42% but was still blocked by GHX, confirming that the signal was generated by SNAT1 (Figure 2G).

### High-throughput assay and compound evaluation

**FIGURE 3:**
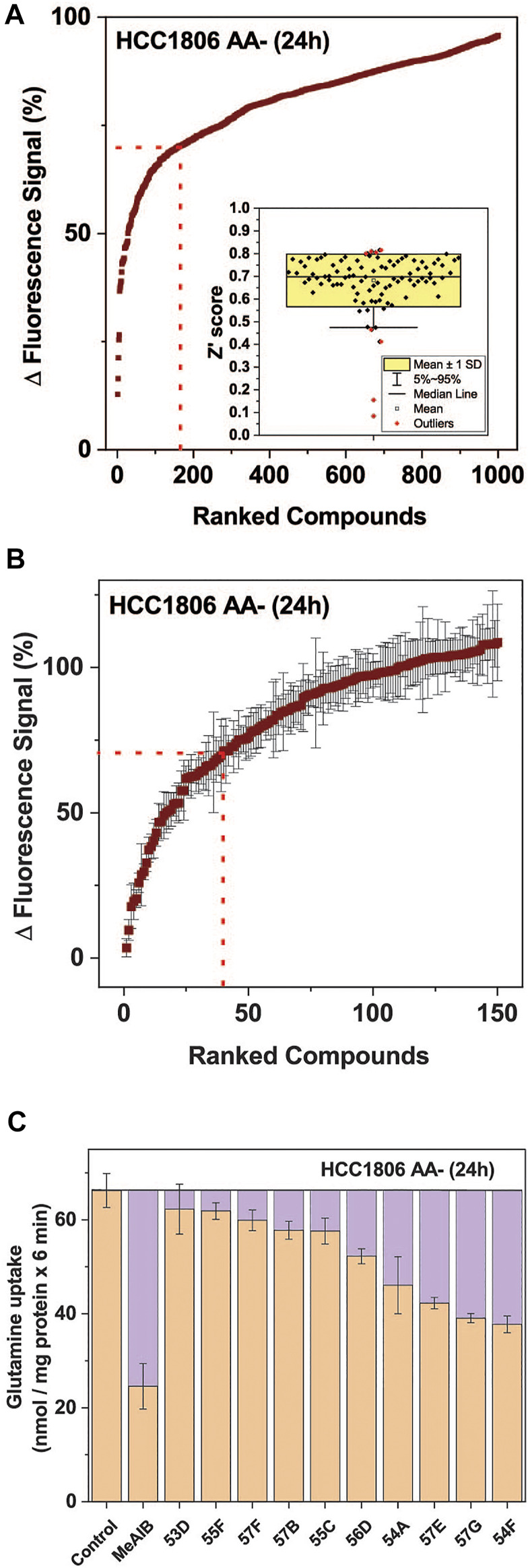
High throughput screen to identify inhibitors of SNAT1 and 2. A total of 33,934 compounds sourced from the Compounds Australia’s Open Collection Scaffolds Library were screened on HCC1806 cells expressing SNAT1 and SNAT2 after amino acid depletion for 24 h. The final concentration of each compound was assayed at 10.05 µM and 1 mM of glutamine was used to stimulate membrane depolarisation. **(A)** The top 1,000 compounds were ranked from most to least potent reduction of the FMP signal. The values shown correspond to the signal change after the injection of glutamine, expressed as a percentage of the baseline reading. The dotted red line indicates the cut-off for compounds included in the confirmatory screen. (Insert) Distribution of the Z′ scores from each of the 384-well plates used in the HTS, calculated from the fluorescence signal changes of negative controls and GHX controls. **(B)** Confirmatory screen of the top 150 compounds. Six replicates for each compound were randomly distributed across three plates and assayed at 10.05 µM. The dotted red line indicates the cut-off for compounds included in the IC_50_ screen, which included 40 compounds. (*n* = 6). **(C)** Ten hit compounds were assayed at a concentration of 50 µM for their effect on the uptake of 100 µM [^14^C]glutamine. The purple boxes represent the extent to which SNAT1/2 were inhibited. MeAIB (10 mM) inhibiting both transporters was used for comparison. The rate from the background control (not shown), where radiolabelled glutamine was incubated in the presence of 10 mM of its unlabelled form, was subtracted. Data are shown as the mean ± STD (n = 3). Abbreviations: MeAIB (N-methyl-aminoisobutyric acid).

Using the optimized assay and HCC1806 cells, which were amino acid depleted for 24 h, a curated diversity library of 33,934 compounds (Open Collection Scaffolds Library, Compounds Australia) was screened at a compound concentration of 10 µM and inhibitors ranked by potency (Figure 3A). Apart from 2 plates, Z’ scores were in a suitable range to identify inhibitors (Figure 3A insert). The top-ranked 171 compounds were selected for a secondary screen (Figure 3B). The top 40 compounds of the secondary screen were then selected for further evaluation in a 9-point dose-response analysis using the FMP assay. Of these, IC_50_-values could be reliably determined for 27 of the assayed compounds (Table 2).

**TABLE 2:** The IC50 of the top 40 hit compounds from the HTS were analysed by FMP assay (*n* = 6).

Assay ID	Vendor	Vendor ID	IC50 (uM)	95% CI
53_A	ChemDiv	6049–0127	10.70	7.872–13.53
53_B	ChemDiv	C169-0201	0.9325	0.5989–1.266
53_C	ChemDiv	M071-0163	N/A	N/A
53_D	ChemDiv	C239-0889	N/A	N/A
53_E	Enamine	Z1341319583	N/A	N/A
53_F	ChemDiv	F687-0643	N/A	N/A
53_G	ChemDiv	C169-0329	1.651	1.224–2.078
53_H	ChemDiv	M071-0172	N/A	N/A
54_A	ChemDiv	F787-0042	20.52	15.82–25.22
54_B	ChemDiv	C273-0133	0.7428	0.5762–0.9094
54_C	ChemDiv	8015–0057	7.108	5.638–8.578
54_D	ChemDiv	C800-0607	5.442	4.791–6.093
54_E	ChemDiv	M071-0030	N/A	N/A
54_F	ChemDiv	M071-0231	11.35	7.631–15.069
54_G	ChemDiv	M071-0138	N/A	N/A
54_H	ChemDiv	8014–2325	4.358	3.689–5.0272
55_A	Enamine	Z90666982	3.836	2.926–4.746
55_B	ChemDiv	L557-0109	6.435	5.888–6.982
55_C	ChemDiv	G327-1596	11.96	7.209–16.71
55_D	ChemDiv	C881-1315	45.35	41.04–49.66
55_E	ChemDiv	E225-0651	N/A	N/A
55_F	ChemDiv	C169-0051	1.424	1.068–1.779
55_G	Enamine	Z1378004448	83.46	24.32–142.6
55_H	ChemDiv	D600-0219	2.419	2.059–2.779
56_A	ChemDiv	C881-0846	4.711	3.960–5.462
56_B	ChemDiv	M071-0113	N/A	N/A
56_C	Enamine	Z31248072	18.44	16.09–20.79
56_D	ChemDiv	F327-0123	3.082	2.210–3.954
56_E	ChemDiv	M071-0406	N/A	N/A
56_F	ChemDiv	8015–0014	7.522	5.617–9.427
56_G	ChemDiv	Z347-0145	N/A	N/A
56_H	ChemDiv	F060-0063	N/A	N/A
57_A	ChemDiv	C169-0373	0.6701	0.5916–0.7486
57_B	ChemDiv	D314-0274	6.806	5.658–7.954
57_C	Enamine	Z254108518	114.3	31.31–197.3
57_E	ChemDiv	L876-0122	10.95	3.605–18.29
57_F	ChemDiv	3236–0188	0.8300	0.6571–1.003
57_G	ChemDiv	V026-9642	7.151	5.806–8.496
57_H	ChemDiv	L221-0185	235.2	130.0–340.4

Fourteen of the 27 compounds were commercially available and their inhibitory potency from the FMP assay was verified using radiolabelled transport experiments, in which the uptake of 100 µM glutamine was challenged by 50 µM of each inhibitor. The radioactive uptake assay constitutes a more direct measure of transporter activity and is considered the gold standard for characterising transport inhibitors (Broer, 2018) (Figure 3C). It was performed on HCC1806 cells after 24 h amino acid depletion. Compounds 54A, 57E, 57G, and 54F inhibited a significant fraction of the MeAIB-sensitive component of glutamine uptake and thus could be considered inhibitors of SNAT1 or SNAT2.

**FIGURE 4:**
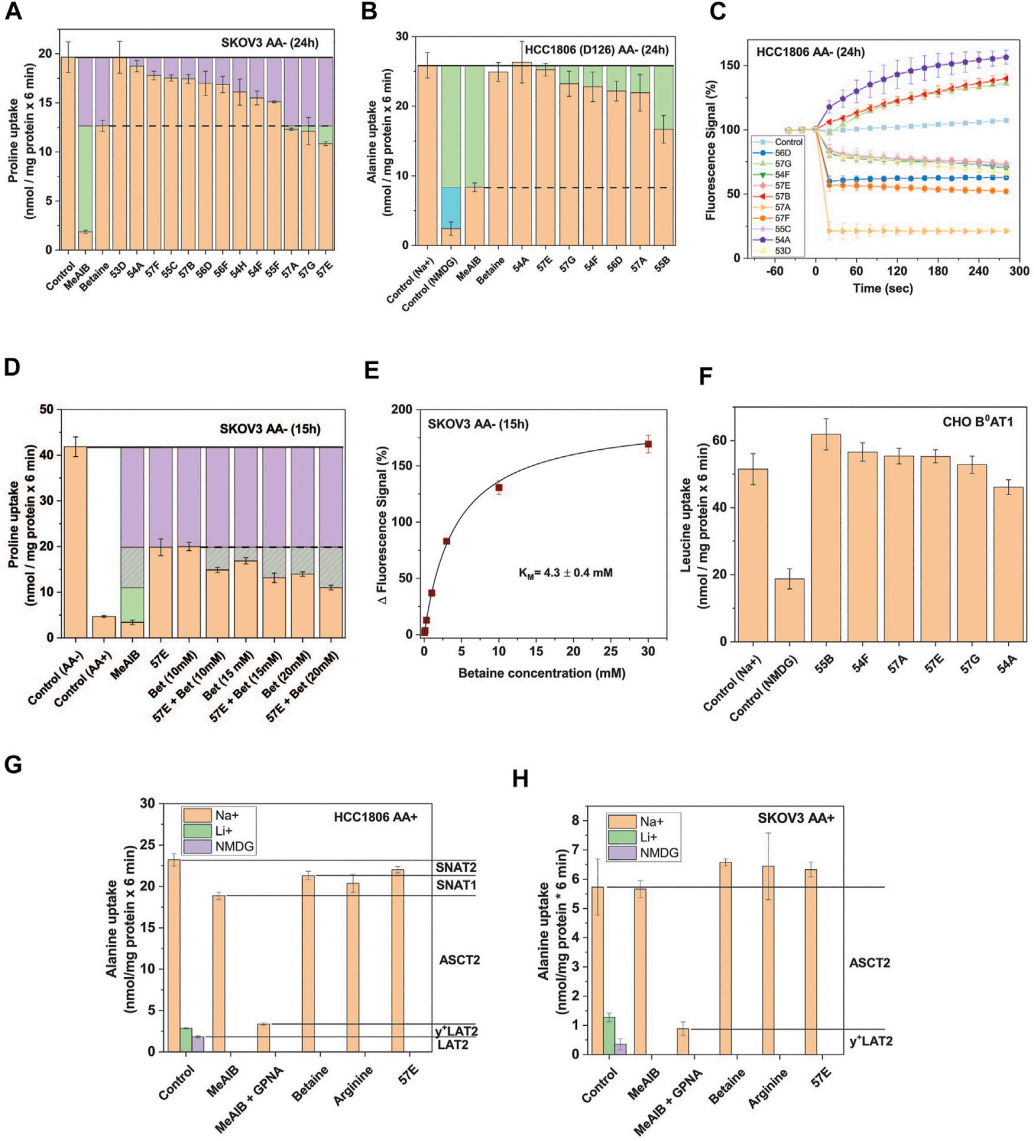
Selective SNAT2 inhibitors identified by HTS. **(A)** Thirteen hit compounds were assayed at a concentration of 30 µM for their effect on the uptake of 100 µM proline in SKOV3 cells pre-incubated in starvation medium for 24 h to induce SNAT2 expression. MeAIB (10 mM) was included to inhibit SNAT1/2, and betaine (10 mM) to selectively inhibit SNAT2. The dashed line indicates the maximum functional contribution of SNAT2 (rates below this threshold signify partial or total inhibition of SNAT1). Green boxes indicate SNAT1 activity. Data are shown as the mean ± STD (n = 3) **(B)** Selected hit compounds were assayed at 50 µM for their effects on [^14^C]proline (at 100 µM) uptake in HCC1806 clone D126 (SNAT2 ko) starved of amino acids for 24 h. MeAIB (10 mM) was included to inhibit SNAT1, and betaine (10 mM) to confirm the absence of SNAT2 activity. Green boxes represent SNAT1 activity and the blue box represents transport activity unrelated to SNAT1/2. The rate from the background control (not shown), where radiolabelled alanine or proline was incubated in the presence of 10 mM of its unlabelled form, was subtracted. Data are shown as the mean ± STD (n = 3). **(C)** HCC1806 cells were amino acid depleted for 24 h to induce SNAT1/2 activity. To determine non-specific effects in the FMP assay, ten hit compounds were introduced to the cells pre-incubated in the FMP dye and mHBSS at t = 0s at a concentration of 30 µM. Upward deflection indicates depolarisation, downward deflection hyperpolarisation. The control consisted of introducing plain mHBSS to the wells. Data are shown as the mean ± STD (n = 3). **(D)** Compound 57E was assayed at 30 µM for its effect on the uptake of 100 µM proline by itself and when combined with increasing concentrations of betaine. SKOV3 cells were pre-incubated in starvation medium for 15 h to induce SNAT2 expression. MeAIB (10 mM) and non-starved (AA+) SKOV3 cells were included as controls to show the upregulation of SNAT1/2 following starvation. The purple boxes correspond to the minimum activity attributable to SNAT2 and the hashed purple corresponds to potential SNAT2 activity. The green boxes represent the activity of SNAT1. The rate from the background control, where radiolabelled proline was incubated in the presence of 10 mM of its unlabelled form, was subtracted. Data are shown as the mean ± STD (n = 3). **(E)** SKOV3 cells were pre-incubated in starvation medium for 15 h to induce SNAT2 expression. Increasing concentrations of betaine were introduced in the FMP assay and the response of the fluorescent signal recorded. Data are shown as the mean ± STD (n = 3). **(F)** Six hit compounds (assayed at 30 µM) were tested in a leucine uptake experiment in CHO cells stably-expressing human B^0^AT1 and its trafficking subunit collectrin. The cells were incubated in sodium-containing mHBSS for 6 minutes with 150 µM leucine in the presence or absence of various compounds. A sodium-free (NMDG) control was included to show endogenous sodium-independent leucine uptake which is not attributable to B^0^AT1. The rate of the background control (uptake of radiolabelled leucine in the presence of 10 mM unlabelled leucine) was subtracted. Data are shown as the mean ± STD (n = 3). **(G)** Uptake of 100 μM [^14^C]alanine was determined in non-depleted HCC1806 cells in the presence and absence of amino acids and their analogues. The contribution of different transporters to alanine uptake was determined by using selective inhibitors as indicated in the margin. **(H)** Uptake of 100 μM [^14^C]alanine was determined in non-depleted SKOV3 cells in the presence and absence of amino acids and their analogues. The contribution of different transporters to alanine uptake was determined by using selective inhibitors as indicated in the margin. Abbreviations: GPNA (γ-glutamyl-p-nitroanilide), NMDG (*N*-methyl-*D*-glucamine), MeAIB (methyl-aminoisobutyric acid).

To elucidate whether the inhibitors were specific for SNAT1 or SNAT2, we measured the uptake of 100 µM radiolabelled proline in amino acid depleted SKOV3 ovarian cancer cells as an independent cellular model for inhibitor evaluation. In this cell line proline is predominantly transported by SNAT1 and SNAT2 indicated by the strong inhibition of proline uptake by MeAIB (Figure 4A). In contrast, betaine inhibited only 30% of proline uptake and was used to discriminate between potential SNAT2 and SNAT1 inhibitors. Three compounds, namely 57A, 57G, and 57E, showed an inhibition equivalent to betaine when applied at 30 µM. To confirm that these compounds inhibited SNAT2 but not SNAT1, we used HCC1806 D126 (ASCT2ko, SNAT2ko) cells (Figure 4B). We used alanine uptake in this experiment, as it is the canonical SNAT1 and SNAT2 substrate, and in this cell clone, cannot be taken up by ASCT2 due to its deletion, thereby reducing background activity. Consistent with the lack of SNAT2, uptake of alanine was not inhibited by betaine. SNAT1 remained active as indicated by the Na^+^-dependence of alanine uptake (i.e. the difference between control (Na^+^) and Control (NMDG)) and its inhibition by MeAIB (Figure 4B). Like betaine, compound 57E did not inhibit alanine uptake, while 57A and 57G showed small effects. To further assess compounds 57A/E/G for unspecific inhibitory effects, we added the compounds at a concentration of 30 µM to the FMP assay in HCC1806 wild-type cells in the absence of amino acid substrate (Figure 4C). Compound 57E generated a small hyperpolarisation (decrease of fluorescence), consistent with blocking the Na^+^ leak current facilitated by SNAT2 (Yao et al., 2000). Compound 57A caused a strong drop of the fluorescent signal, which was considered a confounding factor in the inhibitor analysis. Compound 57G caused a slow depolarisation (increase of fluorescence), which attenuates the electromotive force necessary to drive SNAT2 transport. As a result, we selected compound 57E for further study.

We hypothesized that if 57E was selective for SNAT2 over SNAT1, then combining it with betaine should not exacerbate inhibition of proline uptake in amino acid depleted SKOV3 cells. Consistent with the results in Figure 4A, MeAIB inhibited proline uptake almost completely, while betaine (10 mM) inhibited it by 60% (Figure 4D). Combining both inhibitors increased inhibition slightly, which may reflect incomplete inhibition of SNAT2 by betaine under control conditions. Increasing the concentration of betaine reduced proline uptake further, suggesting that more than 50% of proline uptake in amino acid-depleted cells was mediated by SNAT2. This was confirmed by measuring the K_M_ for betaine, which gave a value of 4.3 ± 0.4 mM (Figure 4E). This suggested that a concentration of 40 mM would be required to fully inhibit SNAT2. However, very high concentrations of betaine might also inhibit SNAT1. To investigate whether the selected compounds could inhibit other electrogenic transporters, we tested their potency against the neutral amino acid transporter B^0^AT1 (SLC6A19) expressed in CHO cells (Yadav et al., 2020). None of the highly-ranked compounds significantly reduced leucine transport via B^0^AT1 (Figure 4F). To further analyse target specificity of compound 57E we tested it on HCC1806 and SKOV3 cells in fresh growth media where the activity of SNAT2 is minimal. Betaine and compound 57E inhibited only about 10% of [^14^C]alanine uptake in amino acid replete HCC1806 cells (Figure 4G). This confirmed the inducible nature of SNAT2 transport and demonstrated that compound 57E did not inhibit any other amino acid transporters in these cells. Gamma-glutamyl-p-nitroanilide (GPNA), a well-known ASCT2 inhibitor (Esslinger et al., 2005), by contrast, inhibited a large fraction of alanine transport. When combined with MeAIB only a small transport activity remained. The remaining contributors to alanine transport were the antiporter LAT2, which remains active when Na^+^ was replaced by NMDG and y^+^LAT2, which remains active when Na^+^ is replaced by Li^+^ and is inhibited by arginine. An analogous analysis of [^14^C]alanine uptake in amino acid replete SKOV3 cells confirmed that compound 57E did not inhibit any transporter in this cell line (Figure 4H). Thus, background activity did not interfere with pharmacological analysis of compound 57E. Overall, the evaluation suggested that compound 57E was a largely selective SNAT2 inhibitor.

**FIGURE 5:**
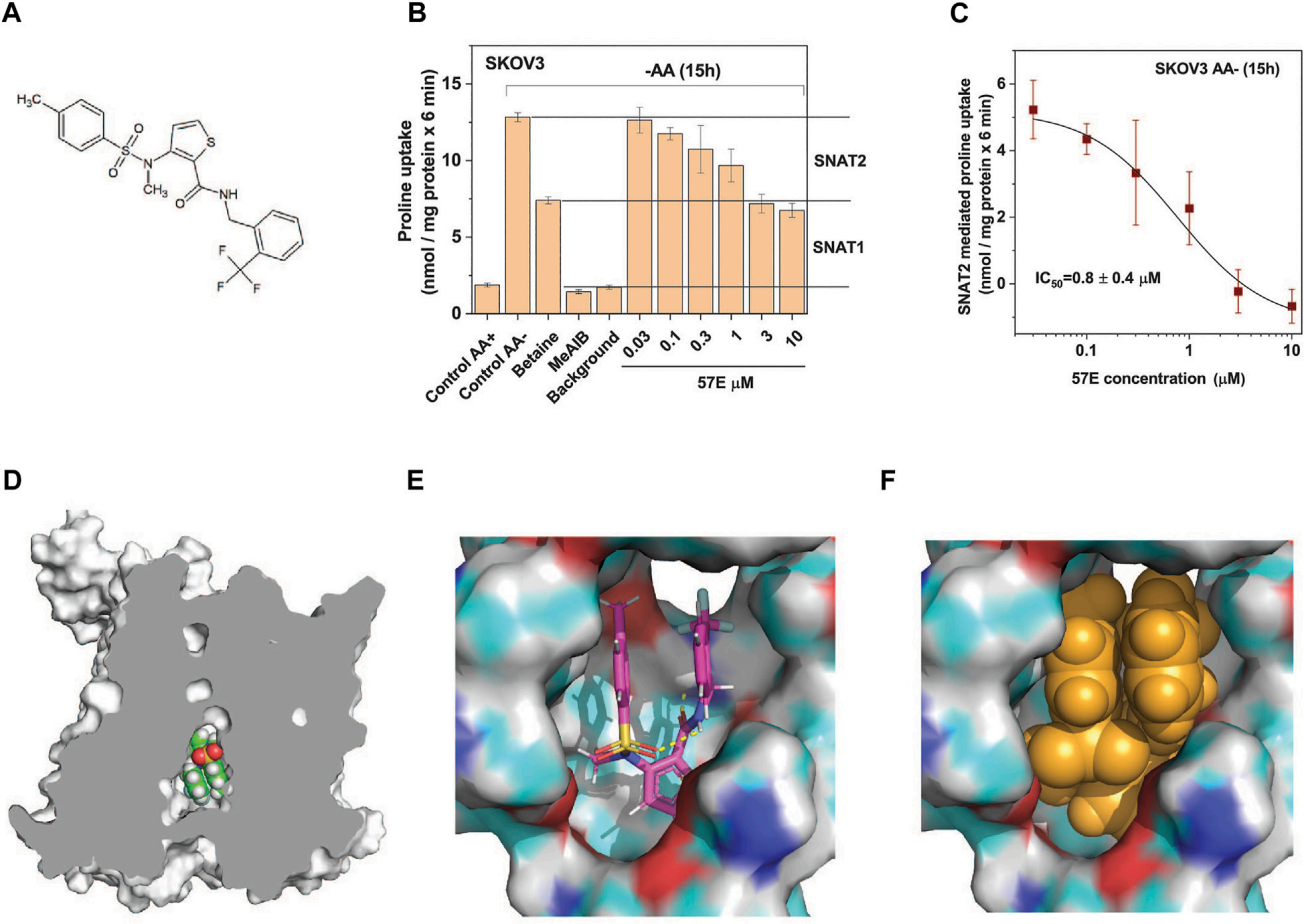
Compound 57E is a high-affinity SNAT2 inhibitor. **(A)** Chemical structure of compound 57E. **(B)** SKOV3 cells were pre-incubated in starvation medium for 15 h to induce SNAT2 expression (control AA-) or remained in full growth medium (control AA+). Compound 57E was assayed at different concentrations for its effect on the uptake of 100 µM proline compared to the effect of betaine (10 mM) and MeAIB (10 mM). Contribution of SNAT1 and SNAT2 to proline transport are indicated in the margin. Background activity was measured as the uptake of radioactive proline in the presence of 10 mM unlabelled proline. **(C)** Data from panel B were used to determine the IC_50_ of compound 57E on proline transport after subtraction of the betaine resistant proline transport. **(D)** Docking of compound 57E was performed using a homology model of SNAT2 based on the structure of *Danio rerio* SNAT9. **(E)** Compound 57E folding up to bind to the cavity of SNAT2. **(F)** Space-fill depiction of compound 57E in the proposed binding site.

Compound 57E (3-(*N*-methyl (4-methylphenyl)sulfonamido)-*N*-(2-trifluoromethylbenzyl)thiophene-2-carboxamide) has a thiophenecarboxamide structure at its centre (Figure 5A). The chemical identity was confirmed by NMR and mass spectrometry (Supplementary Material). A dose response experiment in amino acid depleted SKOV3 cells confirmed that compound 57E blocked the same fraction of transport as betaine (i.e. SNAT2, Figure 5B) and yielded an IC_50_ of 0.8 ± 0.4 µM (Figure 5C) when analysing the betaine-sensitive fraction of proline uptake as a readout signal.

To understand how compound 57E might bind to the transporter we used the Swiss-Model (Waterhouse et al., 2018) homology model of SLC38A2, which is based on the structure of SLC38A9 (Lei et al., 2021) (Figure 5D). Although compound 57E has an elongated structure, docking suggests that it folds up like a sandwich, filling the cavity of the homology model (Figure 5E). The SLC38A9 structure is open to the inside to accommodate a loop of the N-terminus (Lei et al., 2021), which in the homology model is filled by the compound (Figure 5F).

### Biological activity of compound 57E

**FIGURE 6:**
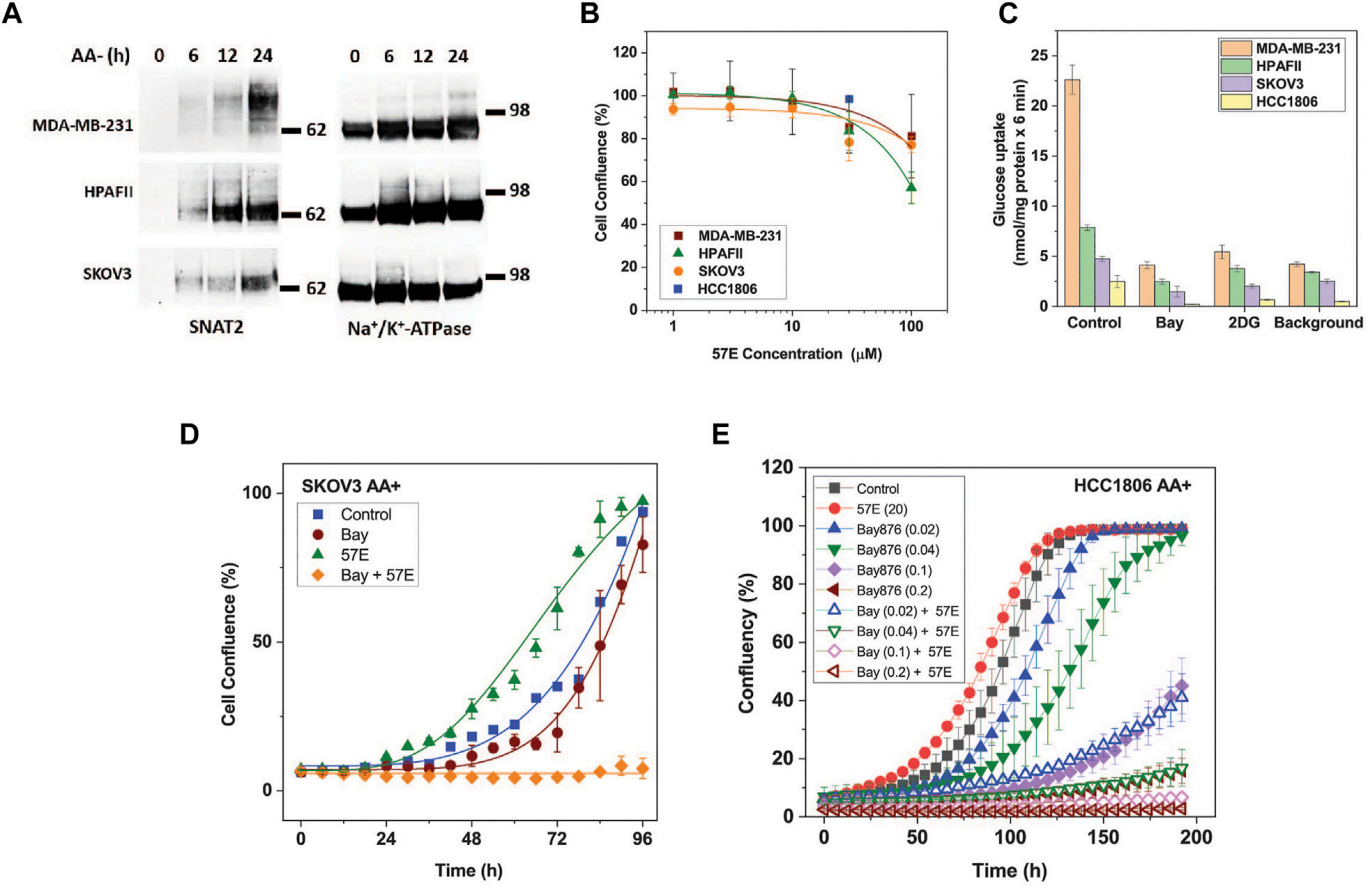
Effect of compound 57E on cancer cell growth. The effect of compound 57E on the growth of HPAFII, SKOV3, HCC1806, and MDA-MB-231 cells was tested to assess its cytotoxic effects. **(A)** Surface expression of SNAT2 was evaluated by surface biotinylation and western blotting in fresh media and after amino acid starvation of up to 24 h. Na^+^/K^+^-ATPase was used as a loading control. For HCC1806 cells see Figure 1A. **(B)** Titration of compound 57E and its effect on growth rates of HPAFII, SKOV3, HCC 1806 (30 μM only) and MDA-MB-231 cells. Cells were seeded onto 96-well plates at 3000 cells cells/well and allowed to attach for 24 h, after which growth medium in the absence and presence of increasing concentrations of compound 57E was added. When control wells reached approximately 90% confluence, each well was analysed by an IncuCyte system to quantify confluence. The mean confluence at various concentrations was normalised to the control and was plotted ± STD (n = 6). **(C)** Glucose uptake in MDA-MB-231, HPAFII, and SKOV3 cells. Cells were incubated with 100 µM [^14^C]glucose for 6 minutes in the presence or absence of 1 µM Bay-876 or 10 mM 2-Deoxyglucose (2-DG). A background control consisting of 10 mM non-labelled glucose was included. Data are shown as the mean ± STD (n = 3). **(D)** Continuous live-cell imaging of SKOV3 cells exposed to 1 µM Bay-876 and 20 µM compound 57E alone and in combination. Logistic growth curves were fitted to the mean confluence of cells at each time point (error bars are ± STD; n = 6). **(E)** Continuous live-cell imaging of HCC1806 cells exposed to different concentrations of Bay-876 and 20 µM compound 57E alone and in combination. Logistic growth curves were fitted to the mean confluence of cells at each time point (error bars are ± STD; n = 4).

Having established that lead compound 57E targeted SNAT2, we assayed its impact on cell growth. The inhibitory potency of 57E was evaluated in HPAFII pancreatic cancer, MDA-MB-231 and HCC1806 breast cancer, and SKOV3 ovarian cancer cells. These cells were selected based on separate clustering with regard to essential genes and oncogenic drivers (Marcotte et al., 2012). All cell lines express high levels of SNAT2 mRNA (log (2)transformed RNAseq counts from the Cancer Cell Line Encyclopedia (Ghandi et al., 2019): HPAFII 7.47, HCC 1806, 6.35, MDA-MB-231 6.54, OVCAR3 7.33, SKOV3 6.26) that are similar to those of LAT1 (SLC7A5 HPAFII 6.99, HCC 1806, 7.12, MDA-MB-231 7.55, OVCAR3 7.65, SKOV3 9.06) an amino acid transporter gene known to be highly expressed in most cancer cell lines. All cell lines showed induction of SNAT2 surface expression upon amino acid depletion (Figures 1A, Figure 6A). To measure growth, cells were seeded out at low density on 96-well plates and incubated with increasing concentrations of compound 57E (up to 100 µM) in growth medium containing amino acids (DMEM/F12/10% FBS/2m M Gln). Growth was compared when control cells reached >90% confluence and the end-point measurement of confluence was recorded for all conditions. All cell lines tolerated compound 57E up to 30 μM, such that an IC_50_ curve could not be plotted within the set concentration range (Figure 6B). Higher concentrations could not be achieved due to the solubility of the compounds. The results showed that compound 57E as a standalone inhibitor did not affect cell growth.

We hypothesised that pairing compound 57E and Bay-876, an inhibitor of the glucose transporter GLUT1, would starve HPAFII and SKOV3 cells of two key substrates required to support cellular energetics and synthesis of building blocks. To measure the functional contribution of GLUT1 to glucose uptake, HPAFII, SKOV3 and MDA-MB-231 cells were incubated with radiolabelled glucose (100 µM) in the presence of Bay-876 (1 µM) and the non-specific GLUT inhibitor 2-deoxyglucose (2DG; 10 mM). Bay-876 inhibited glucose uptake to comparable levels as 2-DG and the background control (10 mM unlabelled glucose) in all cell lines (Figure 6C), demonstrating that GLUT1 was the dominant if not only glucose transporter. Compound 57E (20 µM) and Bay-876 (1 µM) were then incubated with SKOV3 cells separately and together to investigate combinatorial effects. Figure 6D demonstrates that compound 57E alone did not slow growth of SKOV3 cells, however, when combined with Bay-876 it halted growth completely. The same effect was observed in HCC1806 cells, which were quite sensitive to Bay876 alone but even more in combination with 57E (Figure 6E).

**FIGURE 7:**
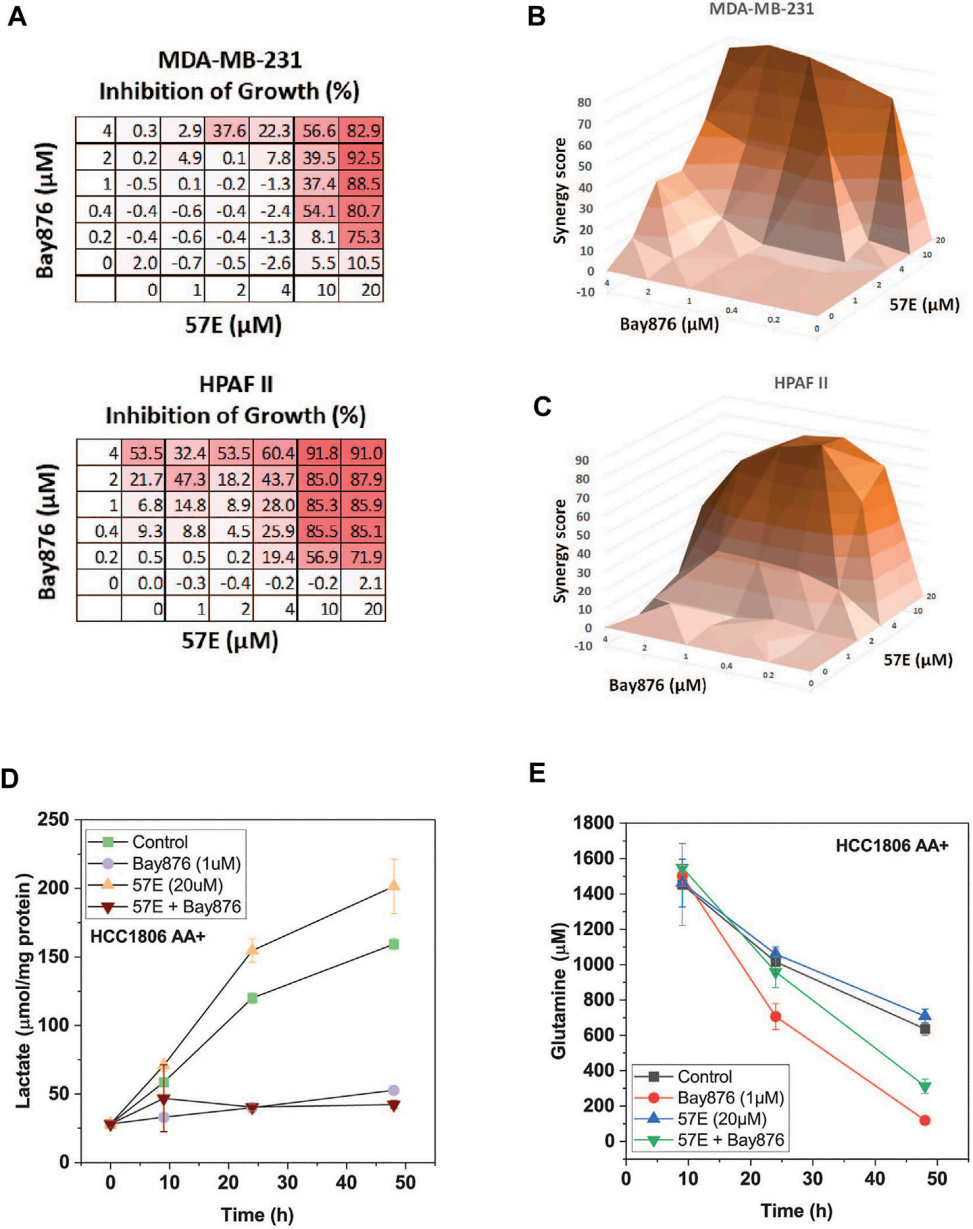
Compound 57E reduces cancer cell growth in synergy with Bay-876. Synergy between Bay 876 and compound 57E was evaluated by monitoring growth of MDA-MB-231 and HPAFII cells in the presence of different concentrations of both compounds. **(A)** Dose-response map of MDA-MB-231 and HPAF2 cells. **(B,C)** Isobologram analysis of MDA-MB-231 **(B)** and HPAFII **(C)** cell growth in the presence of variable concentrations of Bay-876 and 57E. For each combination the growth of the treated cultures was compared to the untreated control cultures once the latter reached >95% confluence. ZIP synergy scores were calculated using SynergyFinder. Averages are shown, (n = 3). **(D)** Lactate production in the supernatant of HCC1806 cells was determined by an enzymatic assay. Cells were treated with Bay-876 (1 μM) and/or 57E (20 μM) for 24 h and samples were analysed at the indicated hours (n = 3). **(E)** Glutamine was determined using LC-MS. Samples were taken from media supernatant of HCC1806 cells treated with Bay-876 (1 μM) and/or compound 57E (20 μM) for 24 h or 48 h (n = 3).

To investigate combinatorial effects in more detail we performed an isobologram analysis in MDA-MB-231 and HPAFII cells. In this analysis both drugs are titrated and cell growth is recorded (Figure 7A). Due to the complex matrices entailed by isobologram analyses we used endpoint assays in which the confluency in the treated condition was compared to the untreated control when the latter reached confluency (>90%). The growth matrix showed that a combination of 2–4 μM of Bay-876 in combination with 20 μM 57E reduced growth by >80% (Figure 7A). To decide whether the combination was additive (i.e. a combination achieves the same as adding more of the same drug) or synergistic (i.e. the combination is more effective than adding more of the same drug) we calculated ZIP synergy scores using SynergyFinder (Zheng et al., 2022) where any value above 0 indicates synergy. Figures 7B,C show the highest synergy when combining 20 μM 57E with 2 μM Bay-876 in MDA-MB-231 cells (Figure 7B) and 10 μM 57E with 0.4 μM Bay-876 in HPAFII cells (Figure 7C).

We have previously shown that SNAT1 and 2 play an important role to provide glutamine for the glutaminolysis pathway (Broer et al., 2016). Oxidation of glutamine through this pathway provides ATP via oxidative phosphorylation. Reducing glutaminolysis by compound 57E increases the burden of glycolysis to provide sufficient ATP. In agreement with this notion lactate production increased in HCC1806 cells upon inhibition of SNAT2 (Figure 7D), while it was blocked completely by > 0.1 μM Bay-876. Vice versa, glutamine consumption accelerated when Bay-876 was used to block glucose uptake (Figure 7E), while compound 57 slowed glutamine consumption when added together with Bay-876.

In summary we show that blocking glucose uptake is synergistic with inhibition of SNAT2 demonstrating a balance between glycolysis and glutaminolysis in cancer cells that can be explored by a novel inhibitor of SNAT2.

## Discussion

In this study, using high-throughput screening of novel scaffolds, we have identified a first-in-class inhibitor for SNAT2, which demonstrates selectivity over the related transporter SNAT1. We deliberately used a strategy to identify inhibitors that are unrelated to the substrate and are less prone to competitive inhibition because screening was performed at elevated substrate concentration. A potential problem in this drug discovery effort was the combined signal generated by SNAT1 and SNAT2. Future HTS could be improved by genomic deletion of SNAT1. However, the present method could identify 3-(*N*-methyl (4-methylphenyl)sulfonamido)-*N*-(2-trifluoromethylbenzyl)thiophene-2-carboxamide (MMTC) as a novel inhibitor of SNAT2.

In the docking studies MMTC always folded like a sandwich, allowing the two aromatic rings to stack up. As a result, a relatively large vestibule is required for binding. Whether MMTC indeed binds to the inside open conformation remains to be shown. The FMP assay includes a 30 min incubation step, which would allow MMTC to diffuse across the membrane. While the docking position is speculative, it opens the possibility that the inhibitor could be useful to study the transceptor function of SNAT2 (Hundal and Taylor, 2009) as it may compete with the binding of the N-terminus to the vestibule of the transporter (Lei et al., 2021).

Functionally, SNAT1 can provide redundancy regarding amino acid accumulation. However, SNAT2 has a unique ability to activate mTORC1, which is not afforded by SNAT1 (Fairweather et al., 2021). The main difference between the two transporters is the higher capacity to accumulate leucine and proline by SNAT2 and its transceptor function (Pinilla et al., 2011), which could potentially be addressed through the use of compounds like MMTC. Proline and leucine play an important role in cancer metabolism as activators of mTORC1, and as factors in stem-cell differentiation and epigenetic control of expression (Tan et al., 2011). SNAT2 is upregulated in response to hyperosmotic treatment, promoting the accumulation of Na^+^ and amino acid osmolytes to maintain cell volume (Bussolati et al., 2001; Menchini and Chaudhry, 2019). Cell proliferation is associated with an increase in cell volume, while apoptosis is associated with cell shrinkage (Lang et al., 2007). Thus, upregulation of SNAT2 could help to maintain an enlarged cell volume to maintain proliferation. This notion is supported by the upregulation of sodium channels, which is frequently observed in cancer cells (Fraser and Pardo, 2008). While SNAT2 activity is hardly detectable shortly after changing media, its surface expression increases as amino acids get exhausted during growth of cells. In the growth assays SNAT2 surface expression will be negligible initially but increases during the growth curve. Moreover, the signaling function of SNAT2 may occur from intracellular compartments (Goberdhan et al., 2016).

Our results suggest that the potential of SNAT2 as an anticancer target could be potentiated by combination therapy. Glucose and glutamine are the main energy nutrients and building block providers for cancer cells and are required to sustain the demands of cancer cells (DeBerardinis and Chandel, 2016). While no clinical trials have been reported for Bay-876, it was shown to be orally available and tolerated in mice at a cancer therapeutic dose (Kopitz et al., 2016). In a more recent study, it reduced the growth of SKOV3 xenografts in mice (Ma et al., 2018), which is consistent with the *in vitro* results reported here for the same cell line. Treatment with Bay-876 did not reduce body weight, suggesting that reduced GLUT-1 activity was well tolerated. However, this study also found that the therapeutic window was narrow and raising the dose from 4.0 mg/kg/day to 7.5 mg/kg/day caused the death of all animals over the 18-days treatment period. The combination of Bay-876 with a SNAT2 inhibitor could improve the therapeutic window.

The basis of the synergistic effect at least in part lies in the balance of energy production between oxidative phosphorylation driven by glutamine and glycolytic ATP production. Inhibiting glutamine uptake via SNAT2 increased lactate production, consistent with a shift of ATP production towards glycolysis. Vice versa inhibition of glucose uptake increased glutamine consumption. This can be counteracted by combination of MMTC with Bay-876.

Combination treatments have been successfully applied to other elements of amino acid homeostasis. Blockade of the cystine/glutamate exchanger xCT (SLC7A11), for instance, works particularly well in combination with drugs that induce elevated oxidative stress (Daher et al., 2019; Lim et al., 2019). Treatment of cancer cells with asparaginase can be augmented by the inhibition of GCN2 (Nakamura et al., 2018), which is a part of the integrated stress response that upregulates amino acid biosynthesis and transport (Ye et al., 2010).

Inhibition of SNAT2 may also be explored in other areas of cellular physiology. Blocking of proline uptake by SNAT2 may prevent development of the embryo before implantation into the uterus (Tan et al., 2016) potentially allowing the development of novel contraceptives. SNAT2 is also involved in transport of alanine into hepatocytes as precursors of gluconeogenesis and may have thus potential to reduce fasting blood glucose levels in type 2 diabetes (Velazquez-Villegas et al., 2021). SNAT2 has also a specific role in the provision of proline to embryonic stem cells (Tan et al., 2011) and osteoblasts (Shen et al., 2022). Blockade of SNAT2 could selectively affect the synthesis of proline-rich proteins such as the transcription factor RUNX2, which have been implicated in cancer cell proliferation and migration (Wang et al., 2013).

While the function of SNAT2 is well understood at a cellular level its systemic role remains to be investigated. No knock-out mouse models have been reported. Generation of specific inhibitors of this transporter open the door to a systematic investigation of its physiological and pathophysiological role.

## Data Availability

The datasets presented in this study can be found in in the Mendeley Data repository: SB; GG-C, AB; MM; AG; RH (2022), “Identification and characterization of a novel SNAT2 (SLC38A2) inhibitor”, Mendeley Data, V1, doi: 10.17632/8p2nxkfvyc.1 The raw data are also available in the article Supplementary Material.
