# Supplementary material for: Identification and characterization of a novel SNAT2 (SLC38A2) inhibitor reveals synergy with glucose transport inhibition in cancer cells
Source: Front Pharmacol. 2022 Sep 21;13:963066. doi: 10.3389/fphar.2022.963066 (PMC9532951; doi:10.3389/fphar.2022.963066)

Chemical validation of compound 57E. The chemical identity of compound 57E, purchased from ChemDiv (cat. # L876-0122), was confirmed using LC-MS and ^{1}H NMR. The purity of the compound was assessed by LC-MS with the correct mass for the proton adduct (calcd. for $C_{21}H_{20}F_{3}N_{2}O_{3}S_{2}$ ($[M+H]^{+}$) 469.0862; found 469.0863). The structure of compound 57E was also confirmed using ^{1}H NMR (700 MHz, $CDCl_3$).

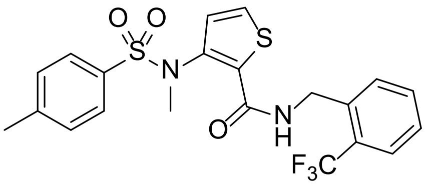

Chemical Formula: $C_{21}H_{19}F_{3}N_{2}O_{3}S_{2}$
Exact Mass: 468.0789

LC-MS 57E, total ion chromatogram, extracted ion chromatogram (*m/z* 469.0863), scan MS (R_t = 14.3 min).

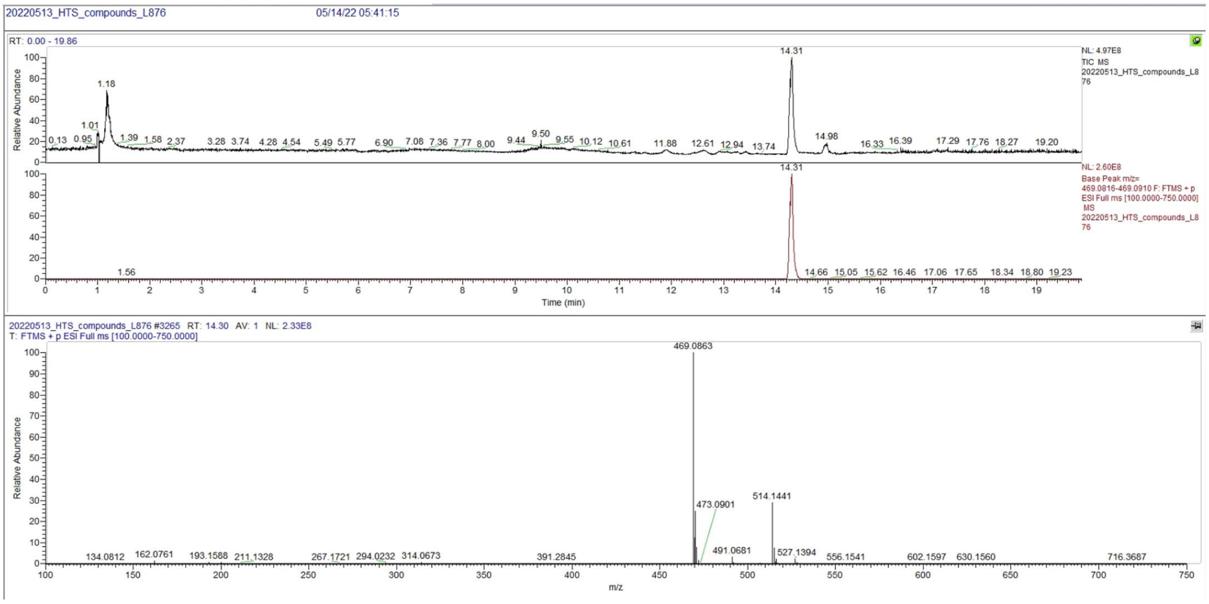

^{1}H NMR (700 MHz, $CDCl_3$) 57E.

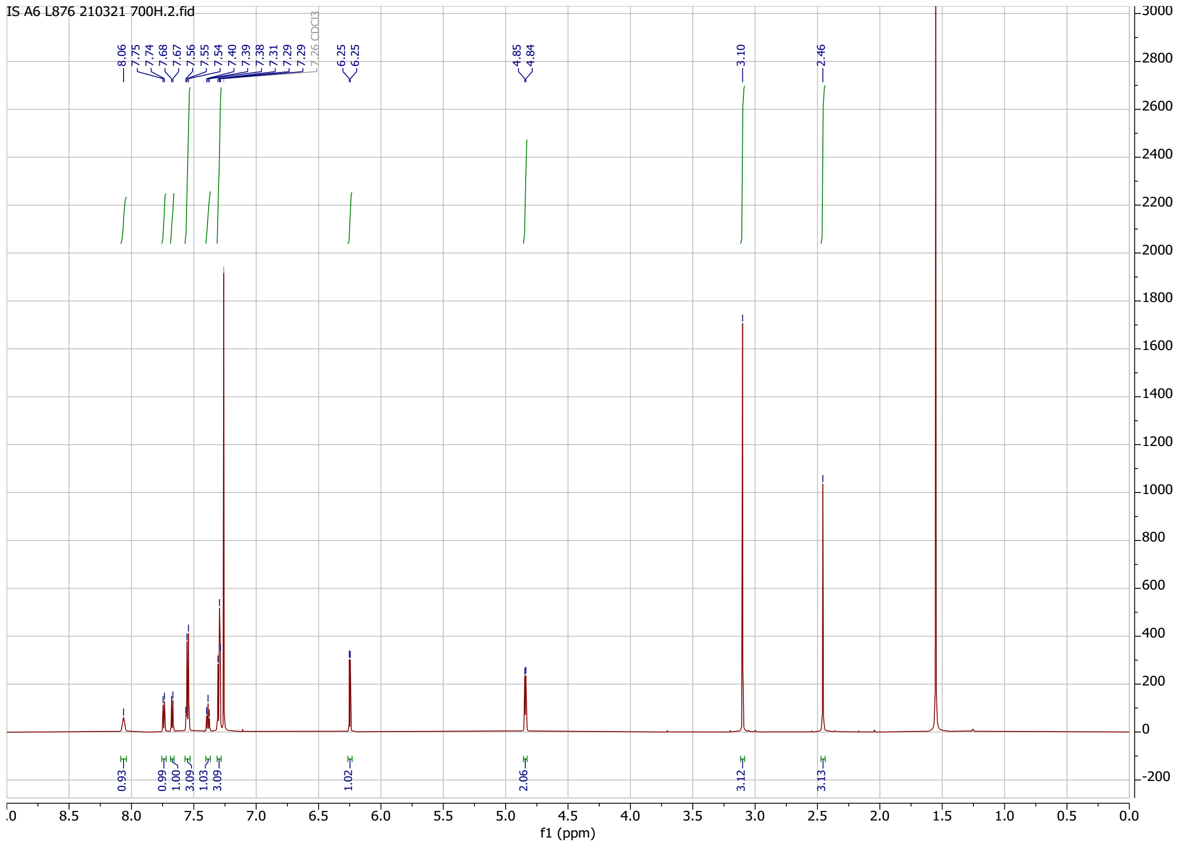

^{1}H NMR (700 MHz, $CDCl_3$) 57E (expanded region).

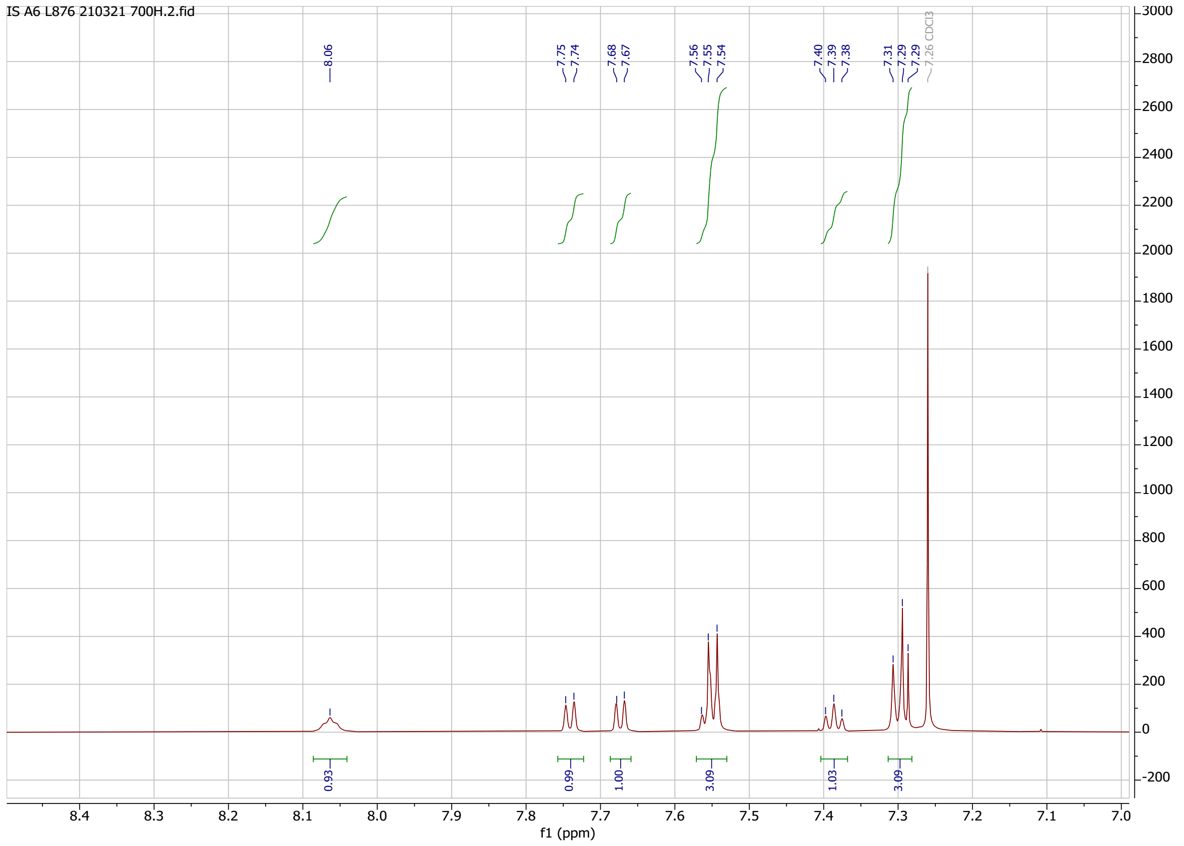

Supplement: Supplementary file 1 [file DataSheet1.PDF]
